# Supplementary material for: Characterization of a Candida albicans Mutant Defective in All MAPKs Highlights the Major Role of Hog1 in the MAPK Signaling Network
Source: J Fungi (Basel). 2020 Oct 17;6(4):230. doi: 10.3390/jof6040230 (PMC7711971; doi:10.3390/jof6040230)
Supplement: Supplementary file 1 [file jof-06-00230-s001.pdf]

Supplementary Materials: The following are available online at www.mdpi.com/xxx/s1, Figure S1: Analysis of gene disruption through Southern blot.; Figure S2: Sensitivity of MAPK mutants to nikkomycin Z.; Figure S3: Yeast-to-hypha transition in 100% serum. Figure S4: Opaque-white phenotype transition of *cek1 cek2 mkc1 hog1* upon incubation at different temperatures.

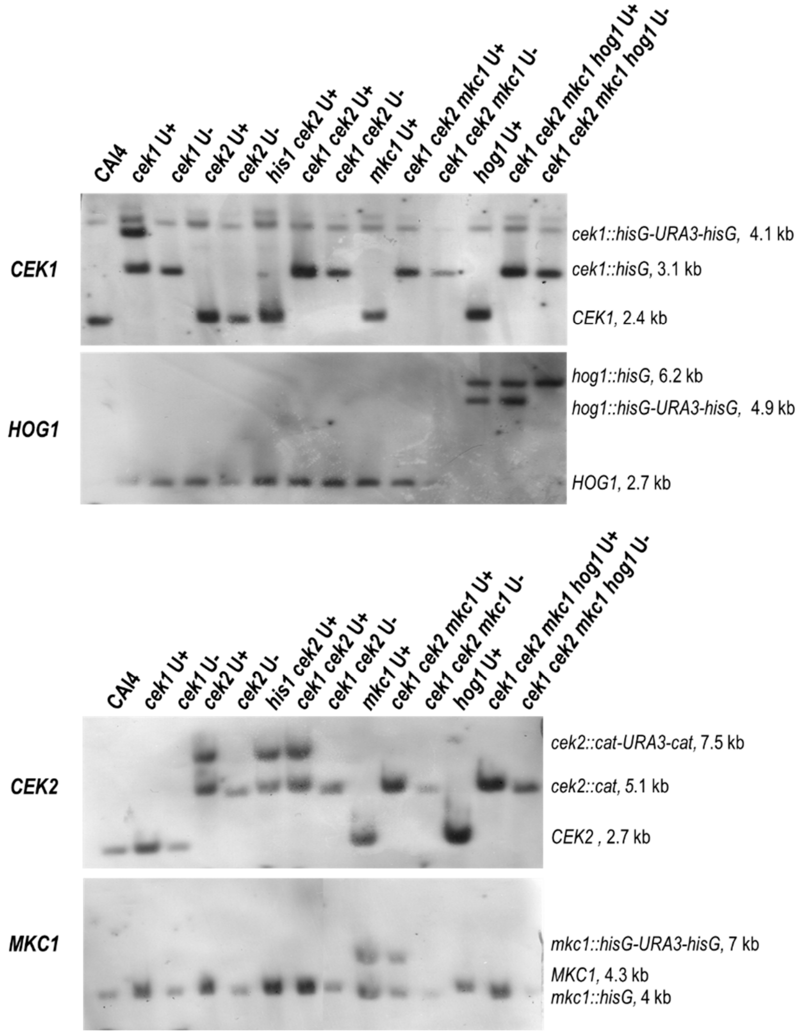

Figure S1. The mutants constructed in this study were analysed through DNA-DNA hybridization to verify gene disruption. In the figure is shown the analysis of the quadruple mutant lineage with four different probes (one for each MAPK). The same genomic DNA was digested with the adequate restriction enzymes (*Hpa*I-*Eco*RV for *CEK1* and *HOG1*, *Sal*I for *MKC1* or *Cla*I for *CEK2*). U+ stands for uracil prototrophy conferred by hisG-URA3-hisG marker; U- stands for uracil auxotrophy upon growth on 5-FOA medium.

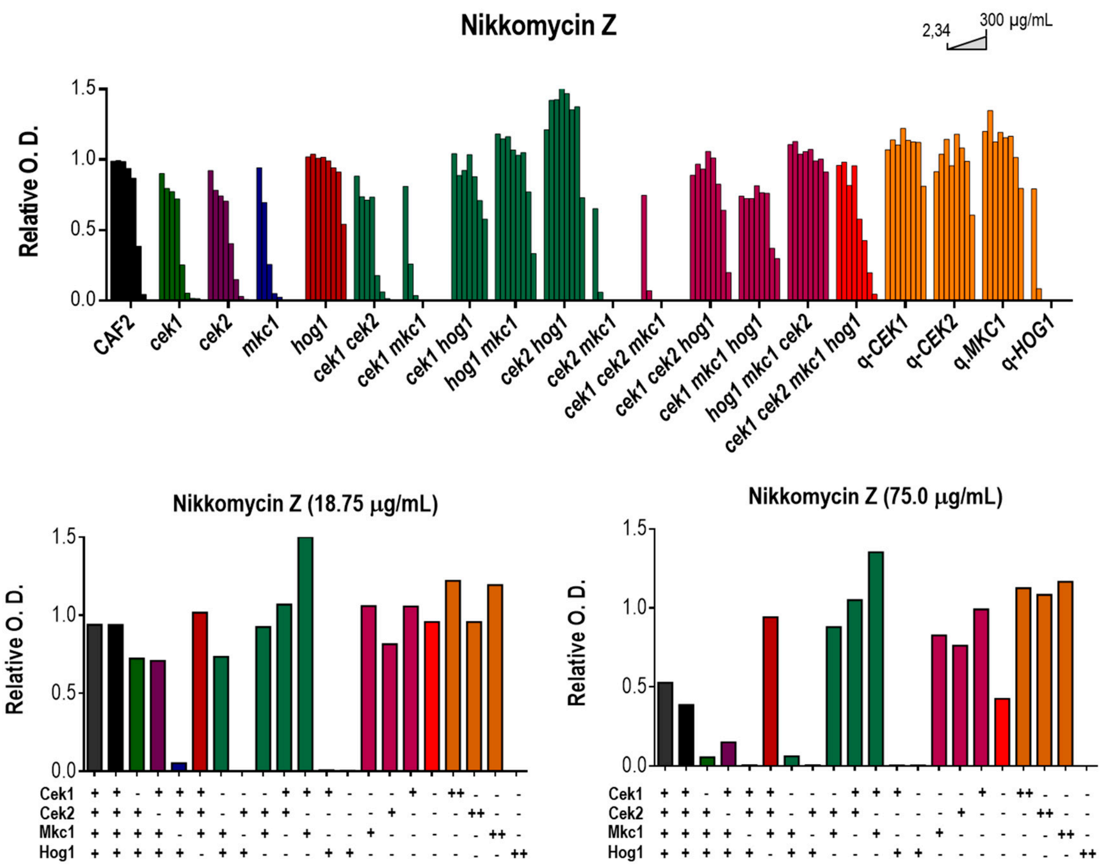

Figure S2. Sensitivity of MAPK mutants to nikkomycin Z. 10^3 cells from overnight grown cultures were inoculated in a 96 well plate with 2 fold increased nikkomycin Z concentrations, ranging from 2.34 µg/mL to 300 µg/mL. The graphics in the lower panel show the relative O.D.s for specific concentrations. The inoculated plate was incubated at 37°C for 24h prior to O.D. assessment. The O.D. profile for each strain was normalized to the positive control (samples without antifungal). + stands for native gene expression, - for gene disruption and ++ for strains expressing ectopically (at *ADH1* locus) the MAPK gene.

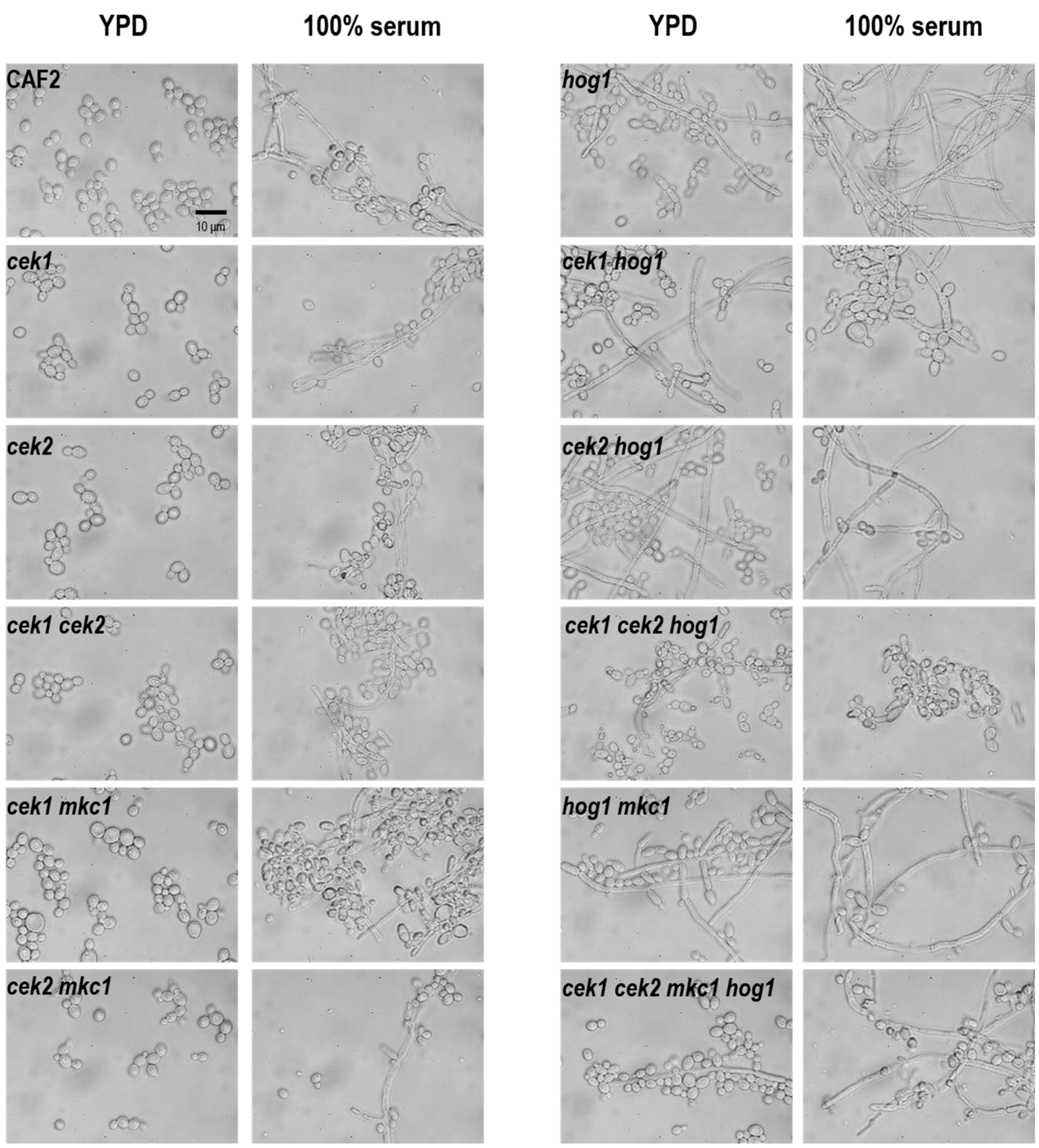

Figure S3. 10^6 cells from overnight cultures were inoculated in YPD medium or 100% serum. Samples were observed after 24h of growth at 30°C and representative pictures are shown.

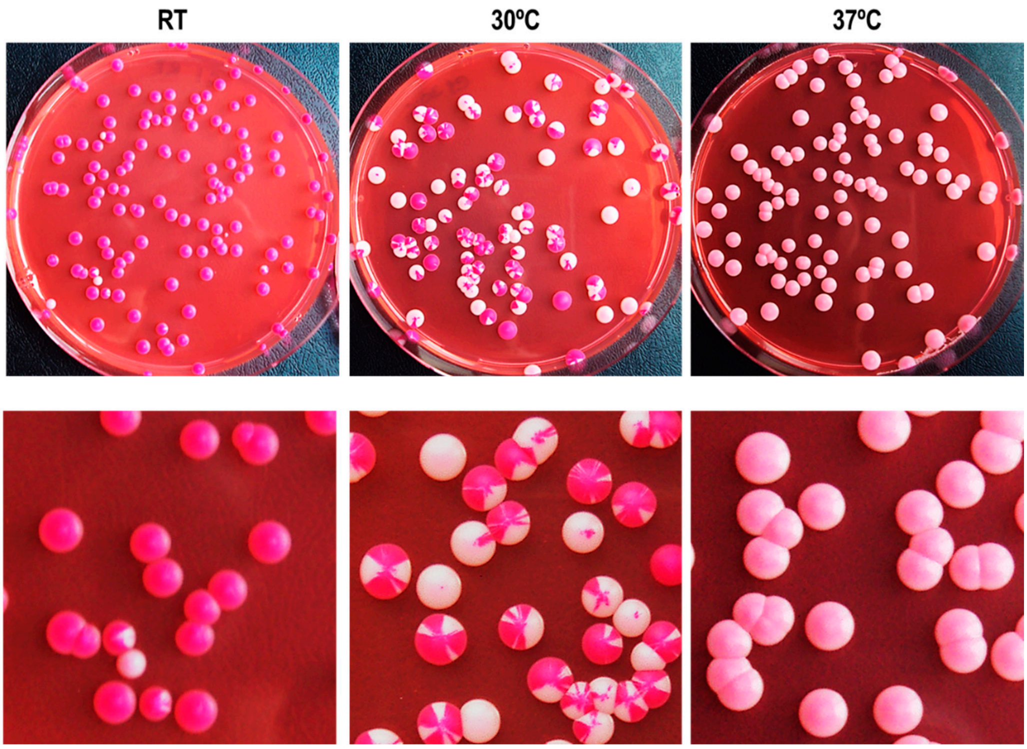

Figure S4. Opaque-white phenotype transition of *cek1 cek2 mkc1 hog1* upon incubation at different temperatures. Opaque cells from a dark-pink colony sector were re-inoculated on YPD (plus phloxine B) and incubated at the indicated temperature. Colonies details are shown on the lower panel.
